# Adaptation and feasibility piloting of a parent-led CBT intervention for youth with anxiety in Czechia

**DOI:** 10.1186/s43058-026-00862-w

**Published:** 2026-02-24

**Authors:** Marie Polášková, Marta Fišerová, Anna Kågström

**Affiliations:** 1 Department of Public Mental Health, National Institute of Mental Health, Klecany, Czech Republic; 2 Department of Psychology, Charles University, Prague, Czech Republic; 3 Department of Global Public Health, Karolinska Institutet, Stockholm, Sweden

**Keywords:** Child mental health, Implementation science, Parent-guided CBT program, Adaptation of an Intervention, Anxiety

## Abstract

**Background:**

Mental health issues in children, particularly anxiety, are a major global concern, with the prevalence of these disorders in Czechia mirroring global trends. While parent-led cognitive-behavioral therapy (CBT) has shown promise in addressing anxiety with benefits like reduced therapist involvement, cost-effectiveness, and long-term sustainability, such programs are scarce in Czechia.

**Methods:**

This study aimed to adapt a Parent-led CBT program from the University of Oxford for the Czech context using complementary frameworks and guides for the process of adaptation to ensure its feasibility, acceptability, and sustainability. Using mixed-methods methodology, we adapted and piloted a parent-led CBT intervention for children with anxiety in Czechia. Adaptations were guided by ADAPT, ToC, CFIR, and TIDieR frameworks to ensure relevant local adaptations. A formative phase included three workshops and 50 semi-structured interviews with key stakeholders to identify barriers, facilitators, and implementation context and needs. Feasibility piloting involved 12 parents, with quantitative outcomes collected via standardized measures and qualitative feedback from interviews and a focus group discussion (FGD) thematically analyzed to inform further implementation adaptations.

**Results:**

The adapted intervention is described having been piloted with Czech parents, yielding promising results: significant reductions in child anxiety, high attendance rates, and positive feedback. This study demonstrated the feasibility and acceptability of the adapted parent-led CBT program for children with anxieties in Czechia.

**Conclusions:**

Guided by ToC and CFIR, adaptations balanced the evidence-based original intervention with culturally and contextually important adaptations to increase future implementation success. Further research should assess effectiveness and implementation fidelity to inform sustainable uptake and integration in the Czech mental health system.

Contributions to the literature

- Research shows that child anxiety is a growing global concern, with few accessible treatment options in Czechia.
- This study tested a parent-led CBT program adapted to the Czech context, which was well-received by parents and led to perceived improvements of the children’s overall well-being.
- These findings highlight the potential of low-resourced parent-led programs and contribute to the understanding of how such programs can be successfully used in different cultural settings, helping to ease the burden on mental health services.

## Background

Anxiety disorders are among the most common mental health challenges affecting children and adolescents worldwide, with nearly 40% manifesting before age 14 [15, 25]. Early intervention can mitigate consequences of psychological difficulties such as poor academic performance and lower life satisfaction [8, 10].

In Czechia, the prevalence of anxiety disorders aligns with global trends; however, psychological support for children and adolescents lacks and there are not sufficient services to meet the mental health needs of the population [28]. Of the 2.2 million children and adolescents in 2023, an estimated 140,000 are affected by anxiety disorders if following estimates consistent with the global prevalence rates [1, 21]. Despite this, access to care is limited, with only 138 outpatient providers in child psychiatry and 90 clinical child psychologists [27].

Parent-led interventions have demonstrated effectiveness in reducing child anxiety by empowering caregivers with skills to support their children’s mental health [17]. These approaches can achieve outcomes comparable to therapist-led treatments while requiring substantially less professional time with approximately 5.5 h of parent training compared to several weeks of individual therapy [26]. Additionally, parent-led cognitive-behavioral therapy (CBT) is more cost-efficient and can be delivered by non-specialists, such as assistant psychologists or trained psychology students, making it a highly scalable and sustainable form of support [5, 26]. While evidence-based parenting interventions for child anxiety exist globally [15] and are routinely implemented in countries such as the USA, Canada, the UK, and Denmark [14], this low-intensity approach is notably absent in Czechia [22].

This study aims to adapt an evidence-based parent-led CBT program developed by the University of Oxford for implementation in the Czech context. The intervention was selected due to its proven effectiveness, brevity, and adaptability for task shifting to non-specialist facilitators [3]. The original program has been used in both individual and group formats [11, 12]. Based on a prior needs assessment in Czechia [22], the group-based format was identified as the most culturally appropriate and feasible. This decision is supported by successful international applications of the group model [9].

Utilizing materials from the individual Online Support and Intervention (OSI) platform and the therapist manual adapted for group-based format [11], this study focused on tailoring the intervention to the Czech context. The program targets parents of children aged 5–12 years and consists of five structured sessions supported by twelve handouts and worksheets. Parents complete mandatory readings between sessions to reinforce learning. Sessions are delivered by two trainers from diverse professional backgrounds, who follow a standardized manual to maintain fidelity while supporting parents in applying CBT skills at home and monitoring child progress [3].

Following cultural adaptation, the intervention was piloted with a small cohort of parents to: (1) assess its appropriateness in the Czech context, (2) understand its mechanisms of action, and (3) explore reasons for variability in outcomes across different settings [19]. This work is guided by implementation science frameworks and guides including the Theory of Change (ToC), ADAPT Guide, Consolidated Framework for Implementation Research (CFIR), and TIDieR, as detailed in Table 1 in the Methods section.

## Methods

The current study was approved by the Ethics Committee of the NIMH on the 20/7/2023, number 132/23. First, we present specific adaptations made to the parenting CBT intervention following the ADAPT (Adapting interventions to new contexts) guide [16], and the feasibility, acceptability and sustainability of the intervention pilot are reported using a ToC [7] and TIDieR (template for intervention description and replication) checklist [13].

### Core intervention

The original parent-led programme [11] aims to teach parents strategies from cognitive behavioural therapy to help their child manage anxiety, using the book *Helping Your Child with Fears and Worries* as a core resource. It consists of five group sessions (8–10 parents) delivered over six weeks, with each two-hour session facilitated by two trained therapists who work collaboratively with parents to tailor CBT principles to their child’s individual needs. Core topics include psychoeducation about anxiety, goal-setting, encouraging independence, using rewards, creating a step-by-step plan, and developing problem-solving skills.

#### 1. Intervention adaptations

Multiple frameworks and tools were used to support an iterative and responsive implementation process, combining a practical guide for systematic and structural adaptations (ADAPT), theoretical and logic grounding (ToC), contextual insight to assess the broader context (CFIR), and rigorous documentation and reporting clarity (TIDieR) (see Table 1 below). The majority of adaptations to content, materials and format were completed prior to piloting, with the pilot phase primarily focusing on testing feasibility and informing adaptations rather than driving major redesign Table 1.

**Table 1:** Integration of frameworks and guides throughout adaptation and piloting process

Framework or guide	Description	Data sources
ADAPT Guide	Provided a staged roadmap for systematically planning and managing the adaptation process, ensuring a structured progression from initial engagement through implementation to evaluation	Project planning and implementation timelines, stakeholder meeting notes, adaptation logs
Theory of Change (ToC)	Clarified the intervention’s multiple interacting components and levels by articulating hypothesized pathways of change, enabling co-design with stakeholders and grounding the adaptation in both theory and local context [7]	Workshops and interviews with key implementers, parents and professionals. Adaptation logs, field notes from facilitation, relevant literature supporting intervention mechanisms
CFIR (Consolidated Framework for Implementation Research)	Informed the contextual inquiry by identifying barriers and facilitators across multiple domains (e.g., inner/outer setting, intervention characteristics), thereby enhancing the relevance and feasibility of implementation	Appendix 1: Parent interview guide exploring format preferences (delivery mode, frequency, format), access to resources and perceptions of helpful support Appendix 2: Czech MH experts' views on program implementation, delivery settings, recruitment, and organizational support Appendix 3: International expert feedback (Iceland) on training quality, implementation challenges, adaptability, support needs (supervision, training of trainers—ToT) and fidelity
TIDieR Checklist	Ensured comprehensive documentation of intervention components and adaptations, enhancing transparency and replicability	Pilot feedback, FGD, revised materials

Three workshops and 50 interviews were conducted with key stakeholders, including parents, trainers, and mental health professionals. All participants provided informed consent. Sessions were led by a professional facilitator, with two researchers (MP, MF) documenting feedback on context-specific adaptations.

### *Adaptation workshops*

Three adaptation workshops were conducted between September 2023 and March 2024 to inform the cultural adaptation and implementation of the intervention. Each workshop addressed specific objectives, including program formation, material adaptation and identification of challenges from the pilot phase. Participants included Czech mental health experts and parents, who provided input on program content and implementation, terminology, visual design, recruitment strategies. Key adaptations included clarifying CBT terminology for accessibility, adjusting materials and visual design, defining recruitment strategies and addressing barriers identified during piloting. Workshop discussions were structured around CFIR domains. Table 2 summarizes the participants, topics covered, aims, and CFIR-based data sources for each workshop.

**Table 2:** Overview of the workshops

Workshop	Participants	Topics covered	Aims	Data sources
Workshop 1 (20/9/23): Program formation and cultural adaptation	11 Czech MH Experts (school psychologists, clinical psychologists, psychotherapists, lecturers in the topic of mental health, a psychologist working with families, and a representative of another parenting program in Czechia)	To brainstorm and answer questions adapted from the CFIR methodology framework [6]	Adapting materials – Gather stakeholder feedback on CBT dictionary Engaging parents – Identify strategies for enrollment and retention Outreach & partnerships – Explore communication channels and key organizations Referral & trainer mapping – Define referral pathways and future trainers	Outer setting: Needs & Resources of Those Served by the Organization - What barriers will the individuals served by your organization face to participating in the intervention? Inner setting: Organizational Incentives & Rewards - What kinds of incentives are there to help ensure that the implementation of the intervention is successful? - What steps should be taken to encourage individuals to commit to using the intervention? Process - What is your communication or education strategy for getting the word out about the intervention? Materials Stakeholders reviewed worksheets to refine mental health and CBT terminology for clarity and accessibility
Workshop 2 (6/11/23): Feedback on materials (design, name, promotional materials, clarity of the translation etc.)	7 Czech parents	Parent brochure (Chapter 1), Worksheets 1 & 2, visual design, program name & logo, recruitment leaflet	Assessment of the appropriateness of translation and content adaptations for the target group Gathering stakeholder feedback on CBT materials Finalise the visual design, including the program's logo and graphics used	Innovation characteristics: Adaptability - What changes or adaptations do you anticipate needing to make for the intervention to be effective in your setting? Design Quality & Packaging - How do you perceive the quality, presentation, and bundling of the intervention materials for implementation?
Workshop 3 (27/3/24): Key questions and challenges identified after the pilot phase	5 Czech MH Experts	Feedback from parents and team members gathered throughout the program piloting (barriers, screening methods, supervision)	To discuss key questions and barriers that emerged throughout the pilot phase for future adaptations, including questions regarding the 1) lack of closure of the intervention, 2) the screening process into the program, and 3) supervision and intervision for the future trainers	Innovation characteristics: Adaptability - What kinds of changes or alterations do you think you will need to make to the intervention so it will work effectively in your setting? Outer setting: Needs & Resources of Those Served by the Organization - What barriers will the individuals served by your organization face to participating in the intervention? Inner setting: Compatibility - Can you describe how the intervention will be integrated into current processes?

### *Stakeholder interviews*

Fifty in-depth semi-structured interviews were conducted across three stakeholder groups: parents, Czech mental health (MH) experts, and international MH experts. Semi-structured Interview Guides (SSIG) for each group included questions adapted from the CFIR framework [6] combined with questions that emerged during piloting (see Appendix 1 for parents, Appendix 2 for Czech MH experts, Appendix 3 for MH experts from Iceland). Semi-structured interviews lasted 35–60 min, guided by the SSIG informed by the CFIR framework which was applied flexibly to capture unanticipated insights as considered good practice in formative research [20]. All transcripts were independently coded by two researchers (MP, MF) using Atlas.ti. Initially, each rater coded the interviews individually and then met to establish the agreed-upon codebook.

Thirty parents were recruited through social media to participate in interviews focusing on three key themes: 1) parental and child worries and their manifestations; 2) perceptions of mental health (MH) and mental health literacy (MHL); and 3) available mental health resources for parents. Only respondents who were parents themselves were eligible to participate in the interviews. Recruitment was not restricted to any specific age group, as the aim of this initial needs assessment was to broadly map parental needs, which had not previously been systematically examined in the Czech context. We acknowledge as a limitation that the parent sample was not limited to the age of children targeted by the intervention (5–12 years). Although the majority of the interview participants were based in Prague, purposive sampling was employed to include parents from diverse regions, especially those with lower SES, such as the Karlovy Vary Region and Usti nad Labem Region. The data from these interviews were coded for themes such as the organizational structure of the parenting intervention.

Additionally, 12 mental health experts from Czechia, including school psychologists, clinical psychologists, psychotherapists, lecturers, a family psychologist, and a representative of another parenting program, were recruited through a snowball sampling method. They provided insights into parental needs, motivation, MH stressors, and barriers to seeking help.

Moreover, 8 international experts were interviewed as representatives of the Icelandic version of the Parent-led cognitive behavioral therapy (CBT) intervention. They provided valuable insights on intervention planning, recruitment, implementation, and challenges. The experts included an associate professor, psychologists, clinicians, and a researcher.

Regular consultations with the original program development team from the University of Oxford further informed the cultural adaptation of the program, emphasizing trainer preparation, screening procedures, and strategies to mitigate dropout rates.

#### 2. Feasibility pilot of adapted intervention

To inform intervention theory, the intervention was piloted with a small cohort of parents, followed by a FGD. Four parents who were available and willing to participate took part in the FGD. As this was a small sample, the findings should be considered preliminary and were used primarily to inform the next phase of the study. All participants provided oral informed consent to participating in the pilot, and the FGD participants provided written consent. During the pilot, field notes were taken by researchers to inform further adaptations and the FGD was recorded and subsequently transcribed with all identifying information anonymized for analysis.

### *Feasibility pilot*

Intervention piloting was conducted from January through February 2024 with a small cohort of parents (*N* = 12) in the Karlovy Vary region. Recruitment was conducted by the study team through outreach activities, including social media posts, flyers and local networking. Parents first self-referred to the programme through an online questionnaire form. Initial screening of participants was based on two questions used in a prior trial [23] to identify elevated child anxiety from a parent’s perspective: (1) ‘*Do fears, worries or anxiety upset or distress your child?*’ and (2) ‘*Do your child's fears, worries or anxiety make things difficult for your family as a whole?*’ Those who screened positive on one or both questions during the initial screening were then contacted for a follow-up by the parent trainers.

This call served to confirm eligibility, assess motivation and availability and ensure a good fit between family needs and the program content. The inclusion criteria for parents to participate was for one parent to commit to full attendance throughout the intervention acting as the primary participant, however all children’s legal guardians/parents were invited to attend all sessions. Most primary parents were mothers (3 fathers participated as primary parents). Parents were instructed to work only with the child experiencing anxiety throughout the intervention, focusing exclusively on the child who qualified for the study via screening, and for whom they also completed the questionnaires and informed consent. The piloting phase was conducted in the Karlovy Vary Region in western Czechia, an area with limited child and family support services. The region’s lower socioeconomic status and lack of care infrastructure made it a relevant setting for testing feasibility and accessibility of the program.

Two parent trainers (a CBT therapist and a therapist-in-training), both previously trained by psychologists from the University of Oxford facilitated the training of trainers (ToT). The inclusion of a therapist in training aligned with the study’s aim to assess whether individuals with less CBT experience could effectively deliver the intervention, based on findings by Thirlwall et al. [26] suggesting comparable outcomes to experienced clinicians. The intervention was structured according to the original group-based protocol, consisting of 2-h weekly sessions over 6 weeks, with a break during the 5th week [11]. The parent trainers also conducted a 4-week follow-up call with each parent. Parents received a self-help brochure which was a compilation of the culturally adapted Online Support and Intervention (OSI) for child anxiety materials which they were asked to follow when completing between-session tasks with their child.

The aim of piloting was to assess the feasibility and applicability of the adapted program in the Czech context via quantitative (questionnaires) and qualitative (focus group) methods. Routine outcome measures (ROMs) were collected before each of the 6 sessions. ROMs included: The Revised Child Anxiety and Depression Scale (RCADS-P), The Outcome Rating Scale (ORS), The Child Anxiety Impact Scale (CAIS-P), The Goal Progress Sheet (GPS), The Group Session Rating Scale (GSRS). Children were asked to complete the following questionnaires both before and after the treatment: The Revised Child Anxiety and Depression Scale (RCADS-C), The Child Outcome Rating Scale (CORS), The Child Anxiety Impact Scale (CAIS-C). For a description of each questionnaire, refer to Appendix 4.

### *Focus group discussion (FGD)*

Following the intervention piloting, the researchers conducted a FGD with 4 parents. Questions were adapted from the Indicative topic interview guide created by international experts from the University of Oxford (See Appendix 5 for the adapted questionnaire). Results from the FGD were used with the stakeholder interviews to inform intervention adaptations.

## Results

### *Participant demographics*

Demographics of participants are described in Table 3 disaggregated across respective stakeholder groups. The median age of children participating was 10.5 (IQR = 5).

**Table 3:** Demographics

Stakeholder group and type of data	Sample characteristics	n	% n
Parents (interviews)	Gender		
	Female	28	93.33
	Male	2	6.67
	Region		
	Karlovy Vary Region	7	23.33
	Prague	9	30
	Central Bohemian Region	6	20
	Usti nad Labem Region	1	3.33
	Hradec Kralove Region	1	3.33
	Vysocina Region	1	3.33
	Zlin Region	1	3.33
	Unknown	4	13.33
Parents (pilot)	Region		
	Karlovy Vary Region	12	100
	Gender		
	Female	10	83.33
	Male	2	16.67
	Child’s gender		
	Female	9	75
	Male	3	25
Czech Mental Health Experts (interviews)	Gender		
	Female	12	100
International experts from Iceland (interviews)	Gender		
	Female	7	87,5
	Male	1	12,5

First, we present adaptations made relating to both intervention components and aspects of program implementation in the Czech context. Second, we present the pilot results from the survey and FGD.

Intervention adaptations were made following the ADAPT guide [16] and are reported in the TIDieR checklist [13], against the original intervention and linked to the justification for each respective adaptation made (see Table 4 below). A ToC map was developed and updated to reflect the process of change within the context of Czechia considering the adaptations. For the implementation adaptation the CFIR framework was used, while intervention components were adapted based on consultations with international experts, intervention piloting and FGD.

**Table 4:** Intervention components and Implementation Process adaptations

Item	Original intervention	Adapted intervention	Why?
WHAT			
Materials	1) Materials for parents: Handouts and Worksheets for each session, 2) Materials for trainers: two-day workshop delivered by the trainer-of-trainers + presentations, Manual	1) Materials for parents: Handouts compiled into a Brochure with additional content on MH literacy, Worksheets compiled into a Workbook, 2) Materials for trainers: Manual and presentations—translation and language adjustments	1) Brochure was created to allow for a more comprehensive and complex knowledge transfer in an in-person setting. A workbook was created based on parental feedback from pilot FGD where parents preferred having all materials in one book 2) Materials were translated with slight language adjustments to accommodate the Czech context
Procedures	1) Recruitment and screening of parents into the program done by clinicians and schools 2) Supervision structure embedded in the clinical setting which allows for regular supervision 3) Battery of Routine Outcome Measures (ROMs) for both individual and group-based intervention includes 5 questionnaires delivered in each face-to-face session (85 questions per session). For the full ROMs, see Appendix 4	1) Recruitment and screening done by involved organizations and individual trainers using a newly developed screening methodology 2) Supervision methodology developed to provide guidance for a supervisor 3) ROMs battery adapted: Session 1 using 3 questionnaires (12 questions) Session 2–4 using 4 questionnaires (15 questions) Session 5 using 5 questionnaires (62 questions) All ROMs were translated, validated and transitioned into an online format	1) Development of screening methodology: A screening methodology with inclusion and exclusion criteria was developed to help trainers select parents who would benefit from the intervention 2) Supervision methodology: as Czechia lacks supervision structures, a methodology was created to guide supervisors of parent trainers to provide support and guidance tailored to the intervention’s needs 3) Adjustment of Routine Outcome Measures (ROMs): based on piloting feedback the length of ROMs was not feasible and therefore were shortened and digitalized
WHO PROVIDED	1) Trainers: Clinical Psychologists (Oxford); Clinicians, Psychologists and Master students (Iceland), Trainer recruitment criteria [4]: Therapists have to work routinely in child mental health services in the UK (most commonly Educational MH practitioners/Trainees, Child well-being practitioners/Trainees, Assistant psychologists). In primary child and adolescent mental health services, the intervention was delivered by Primary Care Mental Health Workers [2] 2) Training: Trainers receive an implementation manual and attend a training day on how to deliver the intervention. The training day includes a mixture of presentations, discussion, group activities and role plays, covering the theory and CBT techniques used in the program	1) Trainers: Social workers, Psychotherapists, School psychologists, Psychologists, Special pedagogists 2) Training: Trainers receive the same training as outlined in the original intervention, ensuring consistency with the established program. Supplementary Training: Additional training was introduced to provide an introduction to CBT, enhancing trainers’ knowledge and skills relevant to the intervention	1) Czechia faces a shortage of child clinical psychologists. Research has shown that the intervention remains effective when delivered by lay professionals Broadening Recruitment Criteria: Professionals, including social workers, school psychologists, and special educators, were recruited to deliver the intervention. Recruitment focused on individuals with experience working with children and parents in mental health or social settings, and prior experience leading group workshops or therapy. This ensured the professionals had the necessary skills to deliver the intervention effectively 2) Enhancing Trainers’ Knowledge Base: CBT principles form the foundation of the intervention. The workshop ensures trainers, from diverse backgrounds like social work and education, gain a consistent understanding of CBT concepts and techniques, enabling effective delivery
HOW	1) Individual Face-to-face intervention (Oxford) + check-in 4 weeks after the 5th session (online/in-person), 2) Online individual program (Oxford), 3) Group-based in-person intervention (Iceland) + check-in 4 weeks after the 5th session (telephone call/in-person)	Group-based in-person mode of delivery. Additional individual sessions after 1 st session (online) and four weeks after the 5th session (online/in-person)	Preference for group-based in-person delivery: Initial interviews revealed that most parents preferred group-based, in-person delivery, which was adopted to enhance engagement Addition of an online goal-setting session: After the pilot and consultations with the international experts, an online goal-setting session was added to address time constraints in the first session
WHERE	Organizations that are involved in the OSI-GROWS project are NHS services, local authorities, third party organizations; local child and adolescent mental health service (CAMHS) [26],Social Services (Iceland)	Social and mental health organisations—community family centres and counselling services, multidisciplinary mental health teams, NGOs, preventative educational care centres, school counselling services	To make the program feasible and available to as many parents as possible, a variety of social and mental health organisations were chosen as collaborative organisations
TAILORING	The final version is not intended to be adapted	The intervention was culturally adapted to the Czech context but the final version is not intended to be adapted	Aspects of the program such as supervision were culturally adapted (see’Procedures ‘) due to different cultural settings in Czechia
MODIFICATIONS		Changes after the pilot (before the testing phase): - Creating a screening methodology - Creating a Supervision methodology - Creating a separate Workbook with worksheets (in the pilot phase parents were given worksheets as separate papers) - Adding an online session after the 1 st session to set goals individually with each parent - Creating a methodology for ToT - Manual—aligning some parts of the text with presentations	See above

### *ToC map*

The Theory of Change (ToC) map for the resulting intervention adapted throughout this formative feasibility and adaptation phase is presented in Appendix 6: ‘Theory of Change Map and Key for the Group-Based Parent-Led CBT Intervention for Youth Anxiety in Czechia’. It illustrates the proposed pathways of change for the adapted group-based, parent-led CBT program, including the intervention’s core components, intermediate processes, and intended outcomes in the Czech context.

### *Pilot of the intervention*

The pilot data showed a promising trend of reduced child anxiety from pre- to post-treatment as evidenced by anecdotal experience of parents. In addition, attendance was high, with 66.67% of parents attending all five sessions. Based on the FGD after the intervention, parents appreciated the supportive atmosphere, practical guidance provided, and normalization of their children's struggles, leading to successfully overcoming children’s fears. For specific feedback from FGD, see Appendix 7.

### Focus group discussion with parents

The FGD aimed to gather feedback on the parents’ experiences with various aspects of the program which resulted in the following key findings:

1. Empowerment of Parents: Parents valued the approach of taking an active role in helping their children, viewing it as both a chance for personal growth and a meaningful alternative to traditional therapy.
2. Preparation and Session Content: Parents found the weekly preparation materials useful, though some initially struggled to grasp certain concepts. More support with goal-setting was recommended, as some found it difficult to define clear, achievable goals.
3. Questionnaires: The questionnaires were viewed as lengthy and cumbersome, with parents suggesting that a more engaging format for younger children (e.g., using pictures) would be beneficial. They also preferred completing the forms online.
4. Program Structure: Parents felt that more time for group sharing and deeper discussion would be helpful. They suggested extending the program or providing additional breaks to allow for reflection and better integration of the material.
5. Positive Impact: Despite challenges, parents reported the intervention had a positive effect on their daily lives, with many expressing satisfaction with the program's overall benefits.

For the results from the FGD with parents after the pilot, see Appendix 7. These FGD findings were used to iteratively update and validate the adaptations made reflected in Table 3 for all aspects related to the complex intervention as defined in ToC (see ToC map).

## Discussion

The aim of the current study was to adapt a parent-led CBT program for the Czech context using a multi-framework approach that combined theoretical structure with flexibility, allowing for iterative integration of stakeholder feedback while preserving evidence-based elements of the core intervention. This facilitated targeted adaptations that balanced fidelity with feasibility, enhancing the program's accessibility and potential for uptake and scale in the Czech context.

By grounding the adaptation process in ToC, we aimed to maintain implementation fidelity while addressing the complexities of the local context, as emphasized by De Silva et al. [7]. To ensure both cultural and practical relevance, the adaptations were systemically guided by the CFIR framework. Key adaptations included intervention materials, implementation procedures, delivery methods, and implementation processes including screening methods and supervisions. These adaptations were informed by a rigorous process of in-depth interviews, focus groups and stakeholder workshops, incorporating insights from Czech parents, Czech mental health experts, and consultations with international experts alongside findings from the piloting phase.

Based on the pilot with parents, preliminary evidence suggests the adapted intervention can improve child anxieties in the Czech context. Attendance rates were high, with 66.67% of parents attending all sessions, and feedback from FGD highlighted key strengths, including supportive atmosphere, practical guidance, and normalization of children’s struggles. These findings align with other studies reporting significant reductions in child anxiety through parent-led interventions [14]. Parents valued their active role in their child's mental health, but identified challenges, particularly in goal setting where additional guidance was needed. While routine outcome measures provided helpful insights, they were viewed as cumbersome by some parents. Suggested improvements included consolidating worksheets into a single structured workbook, extending session lengths, and allowing more time for group discussions. Overall, parents reported positive impacts and offered useful suggestions for refining the program.

Overall, these insights will inform future modifications to improve effectiveness and accessibility of the program. Given the cultural adaptation to the Czech setting, a randomized controlled trial (RCT) with a larger cohort across Czechia is recommended to rigorously evaluate the intervention's effectiveness. Future research should also explore the long-term sustainability of the intervention’s impact, examining whether parental engagement and child anxiety reductions persist over time. Additionally, assessing implementation barriers and trainers’ needs will be essential for ensuring successful integration into Czech mental health services. Finally, future research should formally evaluate the specific contributions of each adaptation to the observed outcomes through fidelity assessments. Lessons from similar adaptations in other cultural contexts, such as parent-led programs implemented in Iceland or Japan, could further inform best practices for scaling up parent-led interventions in non-English-speaking settings [18, 24]. If proven effective, this low-intensity, scalable intervention could help alleviate the burden on mental health specialists, allowing them to allocate resources to children requiring more intensive support for severe anxiety.

## Conclusions

This study successfully adapted and piloted a parent-led CBT program for the Czech context, demonstrating promising results in reducing child anxiety and promoting strong parent engagement. The use of the Theory of Change and CFIR frameworks ensured the program's feasibility, acceptability, and cultural relevance, while maintaining the core principles of the original intervention. Although the pilot findings are encouraging, further refinements are needed to optimize the program. Future research, including a larger randomized controlled trial, will be crucial to evaluate the long-term effectiveness and sustainability of the intervention within the Czech mental health system.

## Data Availability

The datasets for the current study are available from the corresponding author upon reasonable request.

## Appendix 1

### Interview guide for parents

#### Introduction

Children can be a great source of joy, but it may happen that in some situations you as a parent may not know how to help your child in the best way possible. For example, when your child starts to behave differently from what you have been used to, such as withdrawing into themselves, refusing to go to school, feeling very scared, having trouble sleeping or refusing to eat. Such behaviours may be very challenging for parents. In addition, as we are facing a shortage of child psychologists and psychiatrists in Czechia, parents have very limited options where to seek professional help.

At the National Institute of Mental Health, we are currently developing a program for parents that will focus on how you, as a parent, can effectively support your child in overcoming excessive worry. We know from abroad that parenting programs have a positive impact not only on mental health of children but also of parents themselves. The program we are focusing on is originally from the UK and we are trying to adapt it to make it as useful as possible for parents in Czechia.

We would now like to ask you a few questions to help us better understand the specific issues and barriers you face as parents so that we can best design and adapt the program for parents according to your needs. If you have any questions or concerns during the interview, please feel free to interrupt us and ask any questions.

1. Where do parents get information and support in the field of parenting?
  - The format of the program that would suit you best - how often could you be actively involved in the program, how much time could you dedicate to training, how many weeks, where (in what city)? Would you be more comfortable with an online or offline version, group or individual? Would it be possible for you to read materials between sessions? Would you be open to consultations via phone? Who should lead the training according to you? (therapists, school psychologists, psychiatrist, clinical psychologist; men/women; parent/non-parent)
  - Do you have access to a computer and stable internet connection at home? If so, would you have space for an online educational seminar during one evening per week?
  - If there was a book on parenting and how to help your child, would you be interested in it/would you have time to read such a book? Would you prefer a paper or audio version of it?
  - Would you appreciate a book or goodnight stories at home that would help you to start a conversation about worries with your child? Or would you find something like that unnecessary?
  - What kind of parent should promote such a program?

### Appendix 2

#### Interview guide for stakeholders based on CFIR - Czech Mental Health Experts

The questions included: What are MH experts' perspectives on parenting programs and the role of parents in their children's mental health?; Potential barriers faced by participants in the program; What are the needs of parents according to the experience from the expert's work with parents/children?; Cultural adaptations required for the intervention to be effective in the Czech context; What are effective ways to implement the intervention in the Czech context?; What additional materials are needed to ensure the domain of design quality and packaging?; Recruitment process of parents into the program.

**Table:**

Intervention Characteristics Evidence Strength and Quality	What kind of information or evidence are you aware of that shows whether or not the intervention will work in your setting?
	What do influential stakeholders think of the intervention?
	What kind of supporting evidence or proof is needed about the effectiveness of the intervention to get staff on board?
Relative Advantage	How does the intervention compare to other similar existing programs in your setting?
	How does the intervention compare to other alternatives that may have been considered or that you know about?
	Is there another intervention that people would rather implement?
Adaptability	What kinds of changes or alterations do you think you will need to make to the intervention so it will work effectively in your setting?
	Who will decide (or what is the process for deciding) whether changes are needed to the intervention so that it works well in your setting?
	Are there components that should not be altered?
Complexity	How complicated is the intervention?
Design Quality and Packaging	What supports, such as online resources, marketing materials, or a toolkit, are available to help you implement and use the intervention?
Outer Setting Patient Needs & Resources	To what extent is staff aware of the needs and preferences of the individuals being served by your organization?
	What barriers will the individuals served by your organization face to participating in the intervention?
External Policies & Incentives	What kind of local, state, or national performance measures, policies, regulations, or guidelines influenced the decision to implement the intervention?
	What kind of financial or other incentives influenced the decision to implement the intervention?
Inner Setting Implementation Climate Tension for Change	How do people feel about current programs/practices/process that are available related to the intervention? (How do stakeholders feel about parenting programs and the role of parents in their children's mental health?)
Compatibility	Can you describe how the intervention will be integrated into current processes?
	Will the intervention replace or compliment a current program or process?
Relative priority	What kinds of high-priority initiatives or activities are already happening in your setting (Czechia)?
Organizational Incentives and Rewards	What kinds of incentives are there to help ensure that the implementation of the intervention is successful?
	Are there any special recognitions or rewards planned that are related to implementing the intervention?
Goals and Feedback	To what extent are organizational goals monitored for progress?
Readiness for Implementation Leadership engagement	What level of endorsement or support have you seen or heard from leaders?
	What level of involvement has leadership at your organization had so far with the intervention?
	What kind of support or actions can you expect from leaders in your organization to help make implementation successful?
Available resources	Do you expect to have sufficient resources to implement and administer the intervention?
Characteristics of Individuals Knowledge and Beliefs About the Intervention	Do you think the intervention will be effective in your setting?
	How do you feel about the intervention being used in your setting?
Self-efficacy	How confident do you think your colleagues feel about implementing the intervention?
Process Engaging Champions	What kinds of behaviors or actions do you think this individual/champion will exhibit?
Key Stakeholders	What steps have been taken to encourage individuals to commit to using the intervention?
	What is your communication or education strategy (not including training, see Access to Knowledge and Information) for getting the word out about the intervention?
Reflecting and Evaluating	What kind of information do you plan to collect as you implement the intervention?
	To what extent has your organization/unit set goals for implementing the intervention?

## Appendix 3

### Interview guide for stakeholders based on CFIR - International Experts from Iceland

**Table:**

CFIR domains and relevant constructs	Questions	Relevant implementation outcomes
Innovation characteristics	How does the intervention compare to other alternatives that you know about?	Relative Advantage
	Did you have to make any changes or alterations to make the intervention work effectively in the clinical setting?	Adaptability
	What are the most challenging exercises/topics (e.g. step plan, setting goals) of the intervention?	Complexity
Outer setting		
Inner setting - Readiness for implementation	What was the most important aspect of the ToT for you? (Practical aspects/Role play/Theory)	Access to Knowledge & Information
	Would you have appreciated any additional support/information (what was not covered in the ToT)?	Available Resources
	Do you receive any training that helps you tackle group dynamics with parents?	Available Resources, Access to Knowledge & Information
	How does supervision work for you?	Available Resources, Access to Knowledge & Information
Inner setting - Implementation Climate	Did you follow more experienced trainers as part of their training? How did it work?	Learning climate
Characteristics of Individuals	What is your perception of the sufficiency/quality of the ToT?	Knowledge & Beliefs about the Innovation
	Did you feel well-equipped in terms of delivering the intervention following the ToT?	Self-efficacy, Individual state of change
	What are some of the difficulties that come up for you during the implementation process? (lack of self-confidence/lack of knowledge)	Self-efficacy, Individual state of change
Process	How were you assigned to your colleague?	Planning
	Was there anything that surprised you after the ToT in the process of delivering the training?	Engaging, Executing
	What are the key/most important aspects of the collaboration with the second trainer that facilitate the training delivery for you?	Engaging, Executing
	Do you follow the manual step-by-step as it was designed or do you make any ad-hoc changes to accommodate the parental needs in the specific group?	Executing
	What is the average group size that you train?	Executing
	When setting goals with parents - how do you do it in practice? How much support do the parents need? How do you make sure the goals are set correctly?	Executing
	Is there anyone to support the implementation process/evaluate the sessions? Do you get feedback on the training you provide? How/Who provides it to you?	Reflecting & Evaluating
	What are the most common topics that come up for you in the supervision sessions?	Reflecting & Evaluating
Across domains	What are the factors/things that help you deliver the training to parents effectively?	
	Any barriers you encounter that hinder effective intervention delivery? (communication from parents/dropouts/lack of motivation)	
Additional questions	How was it for you to distribute and collect questionnaires from parents?	
	How did parents react to questionnaires?	
	Questionnaire process - Did parents come early to sessions/completed questionnaires online during the session etc.	

## Appendix 4

### Intervention piloting - questionnaires

After the adaptations, the following questionnaires were collected throughout piloting as Routine Outcome Measures (ROMs). The timeline for the assessment of the treatment is presented in Table 7 below.

1. The Revised Child Anxiety and Depression Scale (RCADS-P) measures symptoms of anxiety and depression in children and adolescents. The items assess symptoms of separation anxiety, generalized anxiety disorder, social anxiety, obsessive-compulsive disorder, panic disorder, and depressive disorder.
2. The Outcome Rating Scale (ORS) evaluates the effectiveness of intervention therapy programs. The questionnaire assesses how the parent perceives the child's feelings over the past week in four areas - Personal (personal satisfaction), Relational (family, close relationships), Social (school, friends), and General (overall sense of well-being).
3. The Child Anxiety Impact Scale (CAIS-P) measures how parents perceive the impact of anxiety on the child in school, social, home/family areas, and in general.
4. The Goal Progress Sheet (GPS) is a questionnaire that helps individuals track progress within an intervention. Using this questionnaire during intervention programs allows parents to set goals at the beginning of the intervention (after the first meeting) and monitor progress in achieving these goals.
5. The Group Session Rating Scale (GSRS) evaluates satisfaction with the intervention program and the approach of the training therapists.

Children were asked to fill out the following questionnaires before and after the treatment:

1. The Revised Child Anxiety and Depression Scale (RCADS-C) measures symptoms of anxiety and depression in children and adolescents. The items assess symptoms of separation anxiety, generalised anxiety disorder, social anxiety, obsessive-compulsive disorder, panic disorder, and depressive disorder.
2. The Child Outcome Rating Scale (CORS) is a tool designed to measure the effect of intervention therapy programs. The questionnaire assesses how the child felt over the past week in four areas - Personal (personal satisfaction), Relational (family, close relationships), Social (school, friends), and General (overall sense of well-being).
3. The Child Anxiety Impact Scale (CAIS-C) measures how children perceive the impact of anxiety on their school, social, home/family functioning, and in general.
  **Table 7:** Timeline of the assessment of the treatment
  Session 0 (before treatment)	Session 1	Session 2	Session 3	Session 4	Session 5	After Session 5
  Parents						
  RCADS-P	RCADS-P subscale	RCADS-P subscale	RCADS-P subscale	RCADS-P subscale	RCADS-P	
  CAIS-P	CAIS-P subscale	CAIS-P subscale	CAIS-P subscale	CAIS-P subscale	CAIS-P	FGD
  ORS	ORS	ORS	ORS	ORS	ORS	
  	GSRS	GSRS	GSRS	GSRS	GSRS	
  		GPS	GPS	GPS	GPS	
  Children						
  RCADS-C						RCADS-C
  CAIS-C						CAIS-C
  CORS						CORS

## Appendix 5

### Focus group discussion with parents questions

1. When you learned that you would be attending the meetings and helping your child instead of a psychologist, what were your initial thoughts? Did you have any concerns?
2. How did you find reading the chapters before the meetings and doing the homework during the week? [How much time did you usually spend on reading the chapters and doing homework with your child? Did you refer back to your notes during the parenting program?]
3. How did you feel about the new information and homework assignments? Was it all new to you, or were you already familiar with some of it?
  - Session 1 introduced many new concepts, such as information about anxiety and the vicious circle. Was this information clear and well-explained for you?
4. Regarding questionnaires, could you imagine filling them out on your phone instead of on paper before each meeting? For the questionnaire on services used, were the questions easy to understand? Would you have needed any support in completing them?
5. Was the telephone consultation with the trainers helpful?
6. How could we improve the program? [Suggestions: timing, such as a 3-hour or longer course over 8 weeks; availability of meetings regarding time and location; number of participants; content of sessions].
7. How did other family members, like grandparents or aunts, respond? Did anyone else get involved, or did they make it more difficult?
8. Do you revisit the newly learned techniques after the program, now that it has been two months since the last session?
9. Would you recommend the parenting program to someone you know in the future? [Please share your reasons why or why not, and under what circumstances you would or would not recommend it.]
10. How did you feel about being part of the research study, including completing the questionnaires?
11. If the program were offered like this: "Take part in a research study involving a therapeutic program for parents of anxious children," what information would you need to know to participate in the study?

## Appendix 7

### Focus group discussion with parents results

The aim of the focus group was to gather feedback from parents who took part in the pilot intervention, focusing on their experience of various aspects of the program.

The first part addressed the idea that parents, rather than therapists, should be the ones helping their children. Parents generally mentioned:

- This approach felt positive because it made me realize that as the person in daily contact with my child, I have the power to influence their development. I take it as an opportunity for personal growth as my own progress can help the child. (AV)
- This seemed like a better option since my son, being a boy, wouldn't discuss his feelings with a psychologist, but would with me. It offered us an alternative to move forward and break the cycle we were stuck in. (MP)
- I sought a way to make changes and help my child without needing a psychologist, as my husband was against it. … The major advantage was that I could take action myself, which had a significant positive effect on our entire family. Me attending the sessions proved beneficial for all of us. (ZK)
- I was initially concerned whether my 11-year-old son would cooperate, but I decided to give it a try, believing that even small efforts can eventually make an impact. Although I knew it would require time, I was prepared to invest in it because when you want to help your child, you're willing to sacrifice your own time. (MP)

In terms of the preparation for the weekly sessions (reading chapters and practicing new techniques with children), the following points were mentioned:

- I found it valuable to use the time during the week to process and put in practice what was discussed in the course. Hearing from others and the trainer was fascinating, and I focused on integrating those insights into our own life and challenges. (AV)
- I looked forward to the reading materials, especially after the first chapter was emailed to us. I revisited it often, and as I absorbed more information about anxiety and mental health, everything started to fit together, including the stories that were shared in the group. (ZK)
- I think the preparation is crucial to avoid wasting time during the course. And one can ask questions about unclear points when reading it in advance. It helps them to familiarize themselves with the concepts and techniques. (MP)

Regarding the content of the sessions, parents mentioned the following:

- The terms were new to me. As I read through them, things started to make sense gradually, especially when I began applying them with my child. Some concepts clicked later, even though initially it took me a while to connect the dots, especially with something like the vicious cycle. However, there was nothing I didn't eventually understand. (JP)
- It was new to me as well, but I think it was well-explained. I understood most of it. (MP)

The activity of setting goals was found to be the most challenging and parents generally agreed they needed more support by the trainers:

- If someone had been helpful in assisting us to set and define the goals, it would have been beneficial. I also made my goals too vague at first and only realized later that it wouldn't work like that. Maybe I missed that in the first hour because it went by so quickly. (AV)
- For me as well, I was struggling with it a bit. We set the bar too high and only later realized that we needed to start somewhere completely different… It took me a long time to set those goals, and then I fell behind in filling out the paperwork, and I couldn't catch up anymore (JP)
- I agree that assistance would be beneficial. I set a goal for my son to sleep alone and stop being afraid of the dentist. I don't think we could have resolved these during the course, so we didn't make any progress. It can also be demotivating when you set goals incorrectly and then don't make any progress. (MP)
- After reading that chapter, I had no problem setting goals. However, I named them incorrectly ("Will not be afraid"), and the third one was long-term, which we couldn't achieve and won't achieve for a long time. So, maybe include something we can try out and test in situations we often encounter. (ZK)

When it came to completing the questionnaires, parents generally agreed that the amount of papers was confusing and bothersome and that using their phones to fill them out online would be a more preferable option.

- “However, it's true that later on, we had that graph and summary. That was helpful because I felt like we weren't making progress, but according to those papers, there was subtle movement. It also showed progress in our goals.” (JP)
- “The questionnaire for my daughter was incredibly long and confusing. For young children, it would be beneficial to make it more engaging, perhaps with pictures or emojis instead of numeric scales like 1 to 10. This would help them understand and complete it on their own. Otherwise, it's too lengthy and difficult for a 6-year-old.” (JP)

In terms of improving the program itself, parents mentioned the following points:

- I would prefer having the workbook and the accompanying notebook together. It would be convenient to have all the papers in one place. (JP)
- All participants agreed they would appreciate more time for sharing in the group: “I would appreciate if one meeting was dedicated solely to sharing experiences. It could help participants move forward and exchange tips with each other.” (MP), “It was a bit distracting when the trainer mentioned we needed to hurry due to time constraints. Perhaps having a 1.5-hour session followed by sharing at the end, and setting this framework in advance, would be better.” (AV)

The group for the pilot consisted of 12 parents. Parents agreed the number of participants was appropriate.:

- The 12 stories were perfect, and I really looked forward to them. (JP)
- They were enriching as well. (AV)

Regarding the length of the session and whether it should be spread over more weeks, parents reported the following:

- It was a significant time commitment, but spreading it over more sessions with time for sharing would be beneficial. (ZK)
- When you see the benefits, committing to the 8-week program is manageable. (MP)
- I think the one-week break during the holidays was beneficial. It allowed everything to settle. Having a similar pause in the schedule could be advantageous. (JP)

Additionally, parents have mentioned numerous benefits that the intervention brought into their daily lives.

## Abbreviations

ADAPT: Adapting interventions to new contexts guide
CAIS-C: The Child Anxiety Impact Scale (for children)
CAIS-P: The Child Anxiety Impact Scale (for parents)
CBT: Cognitive-behavioral therapy
CFIR: Consolidated Framework for Implementation Research
CORS: The Child Outcome Rating Scale
GPS: The Goal Progress Sheet
GSRS: The Group Session Rating Scale
FGD: Focus Group Discussion
MH: Mental health
MHL: Mental health literacy
ORS: The Outcome Rating Scale
RCADS-C: The Revised Child Anxiety and Depression Scale (for children)
RCADS-P: The Revised Child Anxiety and Depression Scale (for parents)
ROMs: Routine outcome measures
SSIG: Semi-structured Interview Guides
TIDieR: Template for intervention description and replication checklist
ToC: Theory of Change
ToT: Training of Trainers
